# Enhancement of yogurt functionality by adding *Mentha piprita* phenolic extract and evaluation of its quality during cold storage

**DOI:** 10.1002/fsn3.3981

**Published:** 2024-01-30

**Authors:** Chafika Guemidi, Djamal Ait Saada, Ouiza Ait Chabane, Mahfuz Elmastas, Ramazan Erenler, Mustafa Abdullah Yilmaz, Abbas Tarhan, Salah Akkal, Haroune Khelifi

**Affiliations:** ^1^ Food Technology and Nutrition Laboratory Abdelhamid Ibn Badis University Mostaganem Algeria; ^2^ Department of Biochemistry, Faculty of Pharmacy University of Health Sciences Istanbul Turkey; ^3^ Chemistry Laboratory Gaziosmanpasa University Tokat Turkey; ^4^ Department of Pharmaceutical Chemistry, Faculty of Pharmacy Dicle University Diyarbakir Turkey; ^5^ Department of Chemistry, Faculty of Exact Sciences University of Constantine 1 Constantine Algeria

**Keywords:** antioxidant activity, fatty acids, *Mentha piperita* L., phenolic compounds, yogurt

## Abstract

New functional food products with health benefits are currently in high demand among health‐conscious consumers. The present research aims to improve the functional properties of yogurt by adding peppermint hydroethanolic extract (PHE) at different doses. The impact of PHE (0%, 2%, 4%, and 6%) on yogurt was studied for acidity, pH, organoleptic quality, antioxidant activity, lipid peroxidation, and fatty acid profile. The results revealed that PHE is rich in phenolic compounds, of which rosmarinic acid was the main one (339.88 mg/g lyophilized extract) and has considerable antioxidant potential, which remarkably (*p* < .01) increased antioxidant capacity in yogurt by over 39.51%, even at a low dose of 2%, giving the product better protection against lipid peroxidation and preserving its physicochemical and sensory quality. At 4%, PHE increased significantly (*p* < .01) the content of omega‐3 fatty acids, notably alpha‐linolenic acid, in fortified yogurt compared with the control, and reduced (*p* < .01) the ratio of omega‐6/omega‐3, which dropped from 5.21 to 4.11. It looks feasible to prepare a yogurt with health‐giving properties by adding *Mentha piperita* hydroethanolic extract at a concentration of up to 4% as an alternative to synthetic antioxidants, which would also extend its shelf life.

## Graphical abstract

The phenolic profile and antioxidant activities of *Mentha piperita* hydroethanol extract, as well as its influence on the acidity, pH, antioxidant activity, and lipid profile of a steamed yogurt stored for 20 days at 4°C, were studied. The extract studied is rich in phenolic acids, particularly rosmarinic acid, and has a high antioxidant potential. The addition of *Mentha piperita* phenolic extract considerably increased the antioxidant potential and essential fatty acid content of the yogurt during storage while maintaining an acceptable pH and acidity.

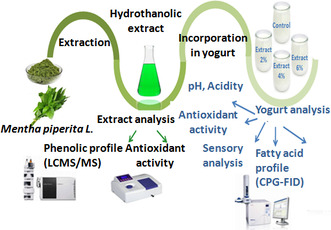

## 1 INTODUCTION

New functional dairy products presenting potential advantages for health are currently in high demand from increasingly health‐conscious consumers (Bulut et al., 2022: Zahid et al., 2023). Among dairy products, yogurt is the most popular fermented milk and an excellent source of protein, calcium, vitamins, lipids, and minerals (Rashwan et al., 2023). However, it is deficient in phenolic compounds (Abdel‐Hamid et al., 2020).

Furthermore, herbs and spices have been used in foods for millennia for flavor enhancement and quality preservation (Kiani et al., 2023). Adding plant matter to yogurt therefore appears to be an effective way of providing natural phytochemicals, which represents a new trend for improving the nutritional characteristics and product functionalities.

Several experiments have also been conducted in this respect to produce yogurts supplemented with bioactive compounds from plants, such as rosemary (Ali et al., 2021) and moringa (Zhang et al., 2019) extracts, Chinese sweet tea (Abdel‐Hamid et al., 2020), and mango peel powder (Zahid et al., 2023). The majority of this research has focused specifically on the antioxidant properties of yogurts supplemented with plant extracts, with only a few going so far as to examine the effects of phenolic extracts on lipid acid composition.

*Mentha piperita* L., also known as peppermint, belongs to the Lamiaceae family and has been widely and traditionally used around the world for flavoring foods (Mahendran & Rahman, 2020) and as a salad (Ayoub et al., 2023). The leaves are also an important component of hot drinks such as Tuareg tea, which is popular in Algeria and North African Arab nations (Benhabyles‐Bouttaba et al., 2018). Other uses of this plant include treating numerous illnesses such as colds, musculoskeletal pain, gastrointestinal tract disorders such as diarrhea, nausea, vomiting, inflammation of the mouth and pharynx, and cramps (Pramila et al., 2012; Trevisan et al., 2017; Valente et al., 2016). Peppermint's key ingredient is its high content of phytochemicals, including polyphenols, making it a highly effective antioxidant for human health compared to synthetic molecules and consequently a major asset for the food industry, given that phenolic compounds inhibit oxidative deterioration in food ingredients, especially lipids, assuring high product quality and enhanced nutritional value (Trevisan et al., 2017).

The present experimental study aimed to use *Mentha piperita* hydroethanolic extract as a functional ingredient in the yogurt and to monitor changes in the physicochemical, organoleptic, and antioxidant characteristics, as well as in the fatty acid composition of the enriched product, during storage at 4°C. The phenolic profile of peppermint hydroethanolic extract and its antioxidant potential were also analyzed in this study.

## 2 MATERIALS AND METHODS

### 2.1 Raw materials and chemicals

*Mentha piperita* L. was harvested in Ouargla, southeastern Algeria, and identified and authenticated at the agronomy department of the University of Mostaganem. Peppermint aerial parts (leaves and stems) were dried in the open air for 15 days at room temperature (21–23°C) without exposure to sunlight and stored in sealed sterile jars protected from light and humidity. The moisture content was determined gravimetrically by drying the dried plant material at 105°C to a constant weight (Karenzi, 2015), and was estimated at 8.77%.

For yogurt preparation, *Streptococcus thermophilus* (*St*) (ATCC 19258) and *Lactobacillus delbrueckii sub sp bulgaricus* (*Lb*) (ATCC 11842), both supplied by SACCO, Italy, were used.

Ethanol (≥99.6%) and chemicals were purchased from Sigma‐Aldrich Co. (Germany). All the reagents and chemicals used were of the highest quality.

### 2.2 Preparation of *Mentha piperita* hydroethanolic extract

10 g of powdered peppermint were macerated in 100 mL of a hydroethanolic solvent (80/20, solvent/water, v/v) for 6 h. Then, the macerate was filtered via Wattman N°1 paper and evaporated using rotavapor (Buchi R‐210 Rotavapor System) at 45°C (Sultana et al., 2009).

The extract obtained was placed in sterilized, sealed glass vials, sterilized by autoclaving at 120°C for 15 min, and then cooled (Ammendola et al., 2020).

The sterility of the extract was confirmed by adding 2 mL of extract to 10 mL of Mueller Hinton. The absence of microbial growth in the broth after incubation at 37°C for 24 h indicates that the extract is sterile.

### 2.3 Phenolic profile and antioxidant activities of PHE

#### 2.3.1 Phenolic profile

Peppermint extract was analyzed with liquid chromatography–tandem mass spectrometry (LCMS/MS) using 56 phytochemical standards. The LC–MS/MS system is composed of a high‐performance liquid chromatography (Shimadzu‐Nexera) coupled to a tandem mass spectrometer (model LCMS‐8040).

Chromatographic separation was achieved using an Agilent Poroshell 120 EC‐C18 column (150 mm × 2.1 mm, 2.7 μm) maintained at a temperature set at 40°C and mobile phases A (water +5 mM ammonium formate +0.1% formic acid) and B (methanol +5 mM ammonium formate +0.1% formic acid) with a flow rate of 0.5 mL/min. The elution gradient used was as follows: 20–100% B (0–25 min), 100% B (25–35 min) and 20% B (35–45 min), making the total run time 45 min per sample. The volume of sample injection was set at 5 μL. The applied mass spectrometry conditions are: drying gas flow (N2) 15 L/min; nebulizing gas flow (N2) 3 L/min; DL temperature 250°C; thermal block temperature 400°C; and interface temperature 350°C (Yilmaz, 2020).

#### 2.3.2 Antioxidant activity

For the evaluation of the antioxidant activity of PHE and the references (quercetin and ascorbic acid), concentrations (3‐2.5‐2‐1.5‐1‐0.5 mg/mL) were prepared in order to deduce the IC50 values required to inhibit 50% of DPPH or ABTS radicals from the inhibition versus sample concentration curves. The absorbance of all prepared extract and reference solutions, as well as that of yogurt serum, was then read using a Janway 7205 spectrophotometer.

##### DPPH‐scavenging activity

100 μL of sample (PHE, quercetin, or ascorbic acid) were added to 2.5 mL of DPPH methanolic solution (1,1‐diphenyl‐2‐picrylhydrazyl) solution (0.04 mg/mL) and incubated for 30 min in the dark. Absorbance readings were taken at 517 nm (Djenidi et al., 2020).

##### ABTS‐scavenging activity

The ABTS+ (2,2′‐azino‐bis(3‐ethylbenzothiazoline‐6‐sulfonic acid)) reagent was initially prepared by mixing in an equal ratio (v/v) aqueous solutions of ABTS (7.4 mM) and potassium persulfate (2.6 mM). The mixture was incubated at room temperature in darkness for 16 h, after which methanol was gradually added until the mixture achieved an absorbance of 1.049 ± 0.012 units at 743 nm. Then, 1800 μL of ABTS+ reagent was added to 200 μL of test sample and incubated for 2 h in a dark room before measuring absorbance at 734 nm (Almusallam et al., 2021).

The negative control, created by substituting the sample with methanol, was treated in the same way as the other samples for both tests (DPPH and ABTS). DPPH or ABTS radical scavenging capacity expressed as a percentage of inhibition was calculated by the following equation:

$$\text{Scavenging activity}\;\left(\%\right)=\left[1-\left({\mathrm{Abs}}_{\text{sample}}/{\mathrm{Abs}}_{\text{control}}\right)\right]\times 100$$

where Abs_Sample_ is the absorbance of the extract sample or standard and Abs_control_ is the absorbance of the negative control.

### 2.4 Preparation of yogurt

#### 2.4.1 Preparation of yogurt fortified with PHE

Cow's milk was supplied by GIPLAIT (an industrial group of dairy products), based at Mostaganem, Algeria, and pasteurized by heating to 90°C for 3 min, then cooled to 45°C and filled into twelve 100‐mL jars. Each batch of three pots received the same dose of 2, 4, or 6% peppermint hydroethanolic extract (PHE), with the exception of the last batch, which served as a control and received no PHE. All milk batches were inoculated with 3% of a St/Lb leaven mix in a 2:1 ratio. Milk pots were sealed, steamed for 4 h at 45°C, and then refrigerated at 4°C (Arioui et al., 2017; Khelifi et al., 2018).

#### 2.4.2 Preparation of yogurt serum

One gram of yogurt was sampled and centrifuged at 9000 *g* for 10 min (4°C) using a SIGMA centrifuge. The supernatants were then filtered using a syringe filter (ISOLAB Laborgerate GmbH, Eschau, Germany) fitted with a membrane (0.45 μm) (Lee et al., 2021). The filtrates obtained were placed at −20°C until further use, preferably no longer than 15 days.

### 2.5 pH and acidity of yogurt

pH and acidity of experimental yogurts were tested according to AOAC (2005) methods. Dornic acidity, given in dornic degrees (°D), was determined by titrating 10 mL of yogurt added with a few drops of phenolphthalein as color indicator, with a 1/9 N solution of sodium hydroxide. The pH was measured using a tabletop pH meter (Benjamin 140 pH meter laboratory, Italy) calibrated with two solutions, acidic and basic.

### 2.6 Antioxidant activity of yogurt

Yogurt serum control and PHE‐supplemented samples were tested for antioxidant activity at days 1, 10, and 20 of cold storage (4°C) by determining the scavenging activity of DPPH and ABTS radicals as described on pages 6 and 5 using the methods of Djenidi et al. (2020) and Almusallam et al. (2021), respectively.

### 2.7 Peroxidation of yogurt lipids

Levels of lipid peroxidation were assessed in experimental yogurts by evaluating the by‐product of lipid peroxidation, malonaldehyde (MDA), by spectrophotometric measurement of the color intensity resulting from the reaction with thiobarbituric acid (TBA) in the presence of trichloroacetic acid (TCA). Briefly, 2 g of yogurt (with 0%, 2%, 4%, and 6% PHE) were taken into glass tubes, which were kept cold by placing them on ice to prevent oxidation reactions. Then, 100 μL of ascorbic acid was added, along with 16 mL of a 5% (w/v) trichloroacetic acid solution. Mixtures were homogenized and centrifuged (SIGMA centrifuge) for 15 min at 8000 *g*, then 2 mL of each supernatant was extracted and supplemented with 2 mL of TBA (20 mmol/L) in glass tubes that were then heated for 15 min in a water bath heated to 100°C and cooled to ambient temperature with cold water. The yellow coloration intensity of each sample analyzed was determined by reading the absorbance at 532 nm using a Janway 7205 spectrophotometer against a blank consisting of 2 mL TBA and 2 mL TCA (Genot, 1996). The absorbance values were converted into mg malonaldehyde equivalent (mg MDA/kg yogurt), using the molecular extinction coefficient of complex TBA‐MDA (1.56.10^−5^ M^−1^ cm^−1^) (Belhadj et al., 2016) according to the following equation:

$$\mathrm{mg}\;\mathrm{MDA}/\mathrm{Kg}=\frac{A_{532}\;\times \;V_{\mathrm{TCA}}\;\times \;72\;\times \;M.10^{5}\;}{1.56\;\times \;m}$$

with *A*_532_: absorbance at 532 nm; *V*_TCA_: volume of TCA (16 mL); M: molecular mass of malonaldehyde (MDA) = 72 g mol^−1^; and m: mass of the analyzed sample (g).

### 2.8 Yogurt fatty acid composition

The fatty acid profile was determined for yogurt formulations with 0% and 4% peppermint extract using gas chromatography with a flame ionization detector (GC‐FID). The technique of Folch et al. (1957), referenced and modified by Karacaglar et al. (2019), was adopted for lipid extraction from yogurt samples at days 1 and 20 of storage at 4°C.

100 g of yogurt were mixed with 200 mL of Folch reagent made up of 2:1 (v/v) chloroform and methanol. After 5 min of magnetic stirring (VELP Scientifica shaker), the mixture was filtered through glass sinter No. 1 (porosity 100–160 μm). The filtrate was mixed with NaCl aqueous solution (0.73%, w/v) at a rate of 1 volume of NaCl to 4 volumes of filtrate, then left to decant into a separating funnel. The lower phase (chloroform + lipids) was collected in a preweighed flask, while the upper phase was rinsed with 50 mL of a mixture composed of 80% (v/v) Folch reagent and 20% (v/v) NaCl aqueous solution (0.58%). The mixture was then agitated and left to decant. The lower phase was racked and added to the previously recovered lower phase, then placed in a rotavapor (Buchi R‐210 Rotavapor System). The total content of fat in 100 mL of yogurt was determined as follows:

TF=W2−W1

where W2 is the weight of the balloon containing lipids and W1 is the weight of empty balloon.

Fatty acid methyl esters were prepared with potassium hydroxide as described by Güneş et al. (2017) and then analyzed by gas chromatography with a flame ionization detector (FID) equipped with a Perkin Elmer Clarus 500 chromatograph and capillary apolar column (RTX‐2330) (USA) (30 m × 0.25 mm and 0.25 μm ID). The carrier gas was helium. The temperature in the oven was adjusted at 70°C for 2 min, then increased at 4°C/min to 120°C, at 2°C/min to 180°C for 3 min, at 4°C/min to 200°C for 3 min and at 7°C/min to 230°C for 5.21 min. The volume of sample injected was 1 μL, for which fatty acids were identified by comparison of retention times with standards, and peak area percentages of compounds were determined using FID data (Demirtas et al., 2011).

### 2.9 Yogurt sensory properties

Yogurt organoleptic characteristics (acid taste, aftertaste, fresher taste, cohesiveness, adhesiveness, smell, and color) were assessed by a panel of 10 participants from the Agronomy Department of Mostaganem University in Algeria, aged between 22 and 54. Yogurts were tested on the first, tenth, and twentieth days of cold storage (at 4°C) and evaluated by panelists according to their preferences on a 10‐point scale, then ranked in order of preference. The results of the ranking test were used to obtain the sum of the ranks.

### 2.10 Statistical analysis

Parametric results from triplicate trials were analyzed statistically using single and two‐level ANOVA variance analysis for total randomization and pairwise means comparison via the Newman–Keuls test. Sensory panel data (from 10 panelists) was processed with the non‐parametric Friedman test. TStatbox 6.4 software was used for statistical processing. Factor effects were assessed at the two probability thresholds of *p* < .05 and *p* < .01.

## 3 RESULTS AND DISCUSSION

### 3.1 Phenolic profile and antioxidant activities of PHE

#### 3.1.1 Phenolic profile

**FIGURE 1:**
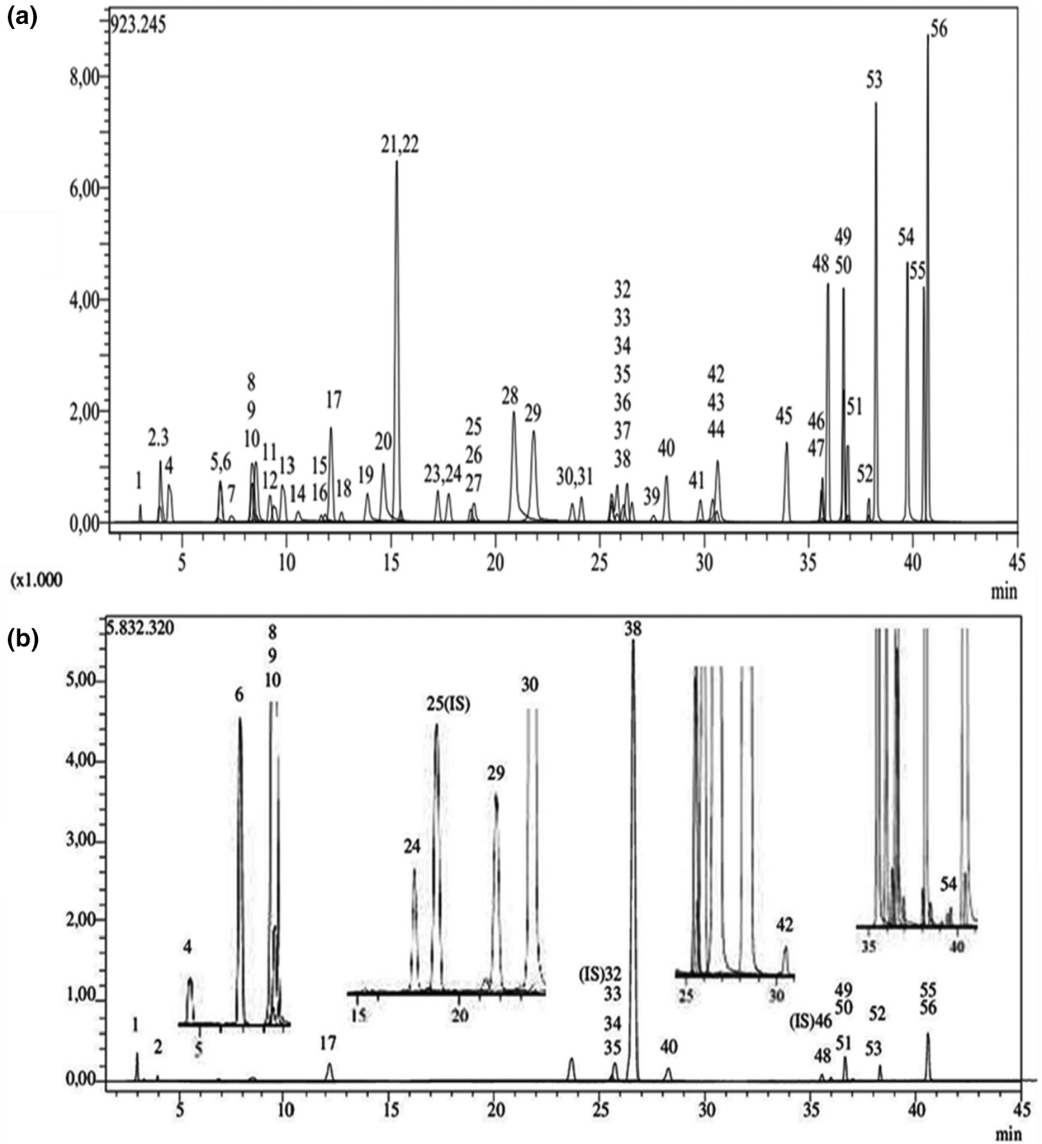
LC–MS/MS chromatograms consisting of (a) TIC (total ion chromatogram) chromatogram of standard phenolic compounds and (b) chromatogram of ethanol:water extract of *Mentha piperita* L. The TIC chromatogram of standard phenolic compounds includes the following compounds, numbered from 1 to 56 (1: Quinic acid, 2: Fumaric acid, 4: Gallic acid, 6: Protocatechuic acid, 8: Gentisic acid, 9: Chlorogenic acid, 10: Protocatechuic aldehyde, 24: p‐Coumaric acid, 29: Salicylic acid, 30: Cyranoside, 33: Rutin, 34: isoquercitrin, 35: Hesperidin, 37: Genistin, 38: Rosmarinic acid, 40: Cosmosiin, 42: Astragalin, 48: Naringenin, 49: Hesperetin, 50: Luteolin, 51: Genistein, 52: Kaempferol, 53: Apigenin, 54: Amentoflavone, 55: Chrysin, 56: Acacetin).

Results reported in Table 1 and Figure 1 showed 26 phytochemical compounds present in PHE detected by LC–MS/MS among 56 standards used, mostly represented by rosmarinic acid, quinic acid, cyranoside, cosmosine, and hesperidin with levels, respectively, of 339.88, 21.03, 11.06, 5.42, and 3.93 mg/g of freeze‐dried extract. Other phenolic acids were also detected in the extract, including caffeic, chlorogenic, protocatechuic, and p‐coumaric acids, respectively, in concentrations of 1.63, 1.50, 0.92, and 0.41 mg/g of lyophilized PHE. Rosmarinic acid's predominance over other phenolic constituents in peppermint extract analyzed by LC–MS/MS is also reported by several authors (Elansary et al., 2020; Kürekci & Beyazit, 2022). This phenolic acid was further identified by Ali et al. (2021) in Australian‐grown rosemary, thyme, mint, oregano, and basil. As one of the most prevalent secondary metabolites of the Lamiaceae plant family, this compound offers a multitude of health benefits, mainly related to its high antioxidant potential as well as its antiseptic and anticancer properties. Its efficacy as an anti‐inflammatory, neuroprotective, and hepatoprotective agent has been confirmed (Hitl et al., 2021; Kiani et al., 2023).

**TABLE 1:** Profile of bioactive compounds in *Mentha piperita* L. hydroethanolic extract analyzed by LC–MS/MS.

Identified compounds		RT (min)	LOD/LOQ (μg/L)^f^	U^g^	Content (mg/g lyophilized extract)
Organic acids	Fumaric aid	03.90	135.7/167.9	0.0091	01.82 ± 0.02
Phenolic acids	Quinic acid	03.00	25.7/33.3	0.0372	21.03 ± 0.78
	Gallic acid	04.40	13.2/17.0	0.0112	00.15 ± 0.02
	Protocatechuic acid	06.80	21.9/38.6	0.0350	00.92 ± 0.03
	Gentisic acid	08.30	18.5/28.2	0.0167	00.05 ± 0.00
	Chlorogenic acid	08.40	13.1/17.6	0.0213	01.50 ± 0.03
	Caffeic acid	12.10	7.7/9.5	0.0152	01.63 ± 0.03
	*p*‐Coumaric acid	17.80	25.9/34.9	0.0194	00.41 ± 0.08
	Salicylic acid	21.80	6.0/8.3	0.0158	00.10 ± 0.02
	Rosmarinic acid	26.60	16.2/21.2	0.0130	339.88 ± 04.42
Flavones	Cynaroside	23.70	12.1/16.0	0.0366	11.06 ± 0.41
	Cosmosiin	28.20	6.3/9.2	0.0083	05.42 ± 0.05
	Luteolin	36.70	2.6/4.1	0.0313	00.90 ± 0.03
	Chrysin	40.50	1.5/2.8	0.0323	00.004 ± 0.00
	Acacetin	40.70	1.5/2.5	0.036	00.81 ± 0.03
	Apigenin	38.20	1.3/2.0	0.0178	00.36 ± 0.01
Isoflavones	Genistein	36.90	3.7/5.3	0.0337	00.05 ± 0.00
Flavanones	Hesperidin	25.80	19.0/26.0	0.0335	03.93 ± 0.13
	Naringenin	35.90	2.6/3.9	0.0392	00.21 ± 0.01
	Hesperetin	36.70	2.6/3.9	0.0392	00.25 ± 0.01
	Amentoflavone	39.70	2.8/5.1	0.0340	00.003 ± 00.00
Flavonols	Rutin	25.60	15.7/22.7	0.0247	00.65 ± 0.02
	Isoquercitrin	25.60	8.7/13.5	0.0220	00.39 ± 0.090
	Astragalin	30.40	6.6/8.2	0.0114	00.07 ± 0.00
	Kaempferol	37.90	10.2/15.4	0.0212	00.06 ± 0.00
Phenolic aldehyde	Protocatechuic aldehydes	8.50	15.4/22.2	0.0396	00.16 ± 0.006

*Note*: Results are expressed as mean values of triplicate samples followed by the corresponding standard deviations (±SD), with a number of replicates *n* = 03; LOD/LOQ (μg/L)^f^, limit of detection/quantification; U^g^ (%), percentage relative uncertainty to 95% confidence level (*k* = 2).

Abbreviations: RT, retention time; SD, standard deviations.

It is undoubtedly largely responsible for the human health benefits provided by *Mentha piperita* L. Our results indicate that other phenolic acids, such as caffeic, chlorogenic, protocatechic, and p‐coumaric acids, are also detected in PHE, respectively, at levels of 1.63, 1.50, 0.92 and 0.41 mg/g of dry peppermint extract. This is greater than the values given by Kürekci and Beyazit (2022), estimating caffeic, chlorogenic, protocatechic, and p‐coumaric acids, respectively, at 0.625, 0.125, 0.363, and 0.194 mg/g extract obtained from peppermint harvested in Turkey and analyzed by ultra‐high‐performance liquid chromatography.

Moreover, in our research, 15 flavonoids, mainly flavones, accounted for 76.83% of all flavonoids detected, the most abundant being cyranoside and cosmosine (11.06 and 5.42 mg/g dry extract, respectively). Flavanones accounted for 18.17% of total flavonoids present in PHE, with high levels of hesperidin, hesperetin, and naringenin estimated at 3.925, 0.248, and 0.212 mg/g dry extract, respectively.

Kürekci and Beyazit (2022) also found that the methanolic extract obtained from peppermint harvested in Turkey contains cyranoside, cosmosine, hesperidin, hesperetin and luteolin at, respectively, 8.55, 8.49, 2.88, 0.14, and 0.56 mg/g. In addition, Elansary et al. (2020) reported that peppermint extract grown in Saudi Arabia contained cynaroside and naringenin at levels of 162.8 and 328.8 mg/100 g dry extract, respectively, analyzed by liquid chromatography with a diode array detector.

It should be noted that a wide range of qualitative and quantitative findings concerning *Mentha piperita* L. phytochemical composition have been reported in the literature, owing to a variety of environmental and/or plant‐related factors such as the physiological state, genetic variation, and evolution stage at the moment of harvesting (Renoz et al., 2022). Extracting, drying, and analysis processes, as well as the harvesting season, have also been implicated (Mahendran & Rahman, 2020; Rohloff et al., 2005).

#### 3.1.2 Antioxidant capacity

Numerous authors have confirmed a favorable association between plant phenolic content and antioxidant function (Ali, Ahmed, et al., 2023; Ali, Cottrell, & Dunshea, 2023; Souilah et al., 2021).

Table 2 shows that PHE exhibits a high capacity for scavenging DPPH radicals, with an IC50 of 3.18 mg lyophilized PHE/mL, which is lower than the values achieved with ascorbic acid (IC50 = 0.16 mg/mL) and quercetin (IC50 = 0.03 mg/mL), but nevertheless considerable, certainly resulting from the detected phenolics, and particularly the major components rosmarinic acid and cynaroside. We also suggest that the presence of water within the solvent composition (ethanol: water; 80:20; v/v) enhanced the antioxidant activity of peppermint extract tested in the present study, leading to an extract richer in phenolic antioxidant compounds, particularly flavonoids, which are water‐soluble antioxidants and are better extracted with the aqueous solvents (Xu et al., 2017).

**TABLE 2:** Antioxidant activity of peppermint hydroethanolic extract compared to that of ascorbate and quercetin.

PHE/standards	IC50 (mg/mL) ± SD	
	DPPH	ABTS
PHE	03.18 ± 0.06	02.49 ± 0.09
Ascorbic acid	00.16 ± 0.02	01.36 ± 0.12
Quercetin	00.03 ± 00.06	01.17 ± 0.18

*Note*: Results are expressed as mean values of triplicate samples followed by the corresponding standard deviations (±SD), with number of replicates *n* = 03.

Abbreviations: IC50, concentration of extract or standard as concentrations inhibiting 50% of DPPH or ABTS radicals; PHE, peppermint hydroethanolic extract; SD, standard deviations.

However, the antioxidant potency of phenolic compounds depends not only on their quantity in the extract but also on their synergistic action. In this regard, it has been demonstrated that binary combinations of rosmarinic acid, caffeic acid, chlorogenic acid, gallic acid, rutin, and quercetin improve antioxidant activity (Hajimehdipoor et al., 2014).

Furthermore, for the same PHE dose, there was a higher IC50 for the DPPH assay (3.18 mg/mL) than for the ABTS assay (2.49 mg/mL). This could be attributable to the presence in the extract of substances with absorption bands at the same wavelength as the DPPH radical, causing higher absorbance (Dieng et al., 2017; Sarr et al., 2015).

### 3.2 Effect of PHE on pH and acidity of yogurt

Table 3 shows values of pH and titratable acidity for plain and PHE‐enriched yogurts over 20 days of storage.

**TABLE 3:** Changes in pH, acidity, antioxidant activity (% inhibition of DPPH and ABTS radicals), and lipid peroxidation levels (TBARS) (mg MDA/kg) of yogurts formulated with peppermint hydroethanolic extract during storage.

Parameters	Storage (days)	Extract incorporation rate (%)			
		0%	2%	4%	6%
pH	1	4.58^e^ ± 0.01	4.77^c^ ± 0.02	4.98^b^ ± 0.01	5.27^a^ ± 0.01
	10	4.45^g^ ± 0.01	4.56^f^ ± 0.01	4.68^d^ ± 0.02	4.77^c^ ± 0.01
	20	4.30^h^ ± 0.01	4.54^f^ ± 0.01	4.67^d^ ± 0.01	4.76^c^ ± 0.01
Acidity	1	80.33^e^ ± 0.01	78.33^g^ ± 0.02	76.33^i^ ± 0.01	70.67^L^ ± 0.03
	10	87.89^b^ ± 0.01	82.44^d^ ± 0.03	78.00^h^ ± 0.05	72.00^k^ ± 0.01
	20	94.48^a^ ± 0.02	87.40^c^ ± 0.02	79.50^f^ ± 0.02	73.39^j^ ± 0.01
DPPH	1	19.36^h^ ± 0.66	58.02^d^ ± 0.99	63.25^b^ ± 0.38	68.55^a^ ± 0.38
	10	15.65^i^ ± 0.32	51.86^f^ ± 0.65	59.68^c^ ± 0.93	62.36^b^ ± 0.23
	20	09.58^j^ ± 0.39	48.96^g^ ± 0.40	49.09^g^ ± 0.24	55.33^e^ ± 0.50
ABTS	1	17.38^h^ ± 0.33	38.52^e^ ± 0.87	51.19^b^ ± 00.05	59.87^a^ ± 0.92
	10	15.67^i^ ± 0.37	31.54^g^ ± 1.59	43.44^d^ ± 00.61	45.68^c^ ± 0.49
	20	12.98^j^ ± 0.42	30.30^g^ ± 0.59	35.20^f^ ± 01.28	36.05^f^ ± 0.56
TBARS	1	0.78^f^ ± 0.05	0.67^g^ ± 0.01	0.58^h^ ± 0.02	0.32^j^ ± 0.04
	10	1.11^e^ ± 0.01	0.72^g^ ± 0.02	0.65^g^ ± 0.03	0.48^i^ ± 0.02
	20	1.52^a^ ± 0.07	1.42^b^ ± 0.01	1.34^c^ ± 0.02	1.20^d^ ± 0.08

*Note*: The results are expressed as mean values followed by the corresponding standard deviations, with a number of repetitions *n* = 03. All factors studied (storage period; extract incorporation rate; interaction of both factors period and extract concentration) show a highly significant effect (*p* < .01); a,b,c,d,e,f: homogeneous groups of two‐to‐two comparison of means according to the Newman and Keuls test.

Abbreviations: D, days; SE, standard error.

Overall, yogurts enriched or not with PHE revealed a significant decrease in pH (*p* < .01) as a function of storage time, from a range of 4.58–5.27 on day 1 to 4.45–4.77 on day 10 and 4.30–4.76 on day 20, in contrast to acidity, which increased significantly (*p* < .01) and proportionally to storage time, from a range of 80.33–70.67 °D on day 1 to 87.89–72 °D on day 10 and 94.48–73.39 °D on day 20 of storage, resulting from the action of starter cultures, whose main role in yogurt production is to acidify the milk by fermenting lactose into lactic acid. At the beginning of fermentation, pH decreases with the growth of *St* making use of amino acids found free in milk or derived from casein hydrolysis, and supplying formic acid and CO_2_ as growth factors to *Lb* (Yamauchi et al., 2019), which grows faster as pH is decreased, releasing more lactate and lowering pH considerably (Chandan & O'Rell, 2013; Naibaho et al., 2022).

According to the scientific literature, yogurt bacteria continue to ferment lactose even during cold storage, producing more lactate (Accolas et al., 1977; Korbekandi et al., 2015; Zourari et al., 1992), particularly by Lb, thus further increasing yogurt acidity (Gallina, 2022). This process, which is called post‐acidification, is considered unfavorable because of its negative influence on product quality, in particular through lactic acid accumulation in the product, which results in an unpleasant taste (Han et al., 2022).

However, the addition of PHE induced a highly significant lowering (*p* < .01) of pH values ranging from 4.44 to 4.62 to 4.78 and 4.93 in mean recorded, respectively, for PHE concentrations of 0%, 2%, 4%, and 6%. On the contrary, titratable acidity values decreased as a function of the extract doses incorporated, ranging from 87.57 to 82.72 to 77.94 and 72.02 °D in mean values obtained for yogurts with 0%, 2%, 4%, and 6% extract. This could be due to the inactivation of viable bacteria in yogurt or a reduction in count resulting from the phenolic compounds provided by the added PHE, whose antibacterial effect has been confirmed (Wei et al., 2023). Similar trends were observed for yogurt fortified with different doses of rosemary extract (Ali et al., 2021). Furthermore, Khelifi et al. (2018) demonstrated the antibacterial effect of phenolic extract from Lamiaceae plants, specifically *Thymus vulgaris* L., against *St* and *Lb*, as well as an increase in pH accompanied by a decrease in yogurt acidity as a function of the doses of phenolic extract added during both the fermentation and storage periods. Phenolic compounds exert their antibacterial effects through different modes of action: by damaging the cell walls of bacteria, causing the loss of cellular constituents; by blocking the production of cellular energy; or by destroying the genetic material of bacteria (Yang et al., 2008).

In contrast, some other authors reported no significant effect on pH values or acidity by adding certain herbal extracts, such as moringa (Zhang et al., 2019) and Chinese sweet tea (Abdel‐Hamid et al., 2020), to yogurt preparation. This divergence of results suggests that the effects of adding plant extracts to yogurt acidity and pH levels, which are closely linked to the viability of starter bacteria, depend on the phenolic content of the added extract, to which plant species, plant physiological states, and environmental factors all contribute (Renoz et al., 2022).

However, it is clear that throughout the storage period, the acidity values measured in plain and fortified yogurt were well within the normal range of 60–150 °D, corresponding to a lactic acid concentration that ranged between 0.6 and 1.5 g per 100 g of yogurt, as reported by de Moura et al. (2019).

### 3.3 Effect of PHE on the antioxidant activity of yogurt

As indicated in Table 3, yogurts with PHE supplementation showed significantly (*p* < .01) greater antioxidant activity (AA) compared to plain yogurt over the period of cold storage.

At a low dose of just 2% PHE extract, DPPH radical scavenging activity in enriched yogurt increased by 38.66% and 39.38% over the control, respectively, on the first and 20th days of cold storage, while it increased significantly by around 49.19% over the control at a concentration of 6% PHE on the first day of refrigeration. Similarly, the ABTS scavenging activity of yogurt added with PHE at 2% was enhanced by 21.14% and 17.32% in comparison with the control on days 1 and 20, respectively, and by 42.49% on day 1 when prepared with 6% PHE.

It has been reported in the literature that the AA of yogurt is partly attributed to the bacterial metabolic activity of its bacteria, which produce organic acids and bioactive peptides (Cho et al., 2020). In our study, the results reveal a possibility of improving yoghurt AA by adding PHE, which contains a variety of phenolic antioxidant compounds, as mentioned above, mainly rosmarinic acid, renowned for its high antioxidant potential (Elansary et al., 2020), but also caffeic acid, cyranoside, cosomosin, and many other compounds.

These results correspond to those revealed by Cho et al. (2020) and Mohamed Ahmed et al. (2021), who found that incorporating phenolic extracts of olive and argel leaves increased the AA of yogurt.

Furthermore, as storage time increased at 4°C, all experimental yogurts showed a decrease in DPPH trapping activity (from 19.36%, 58.02%, 63.25%, and 68.55% on day 1 to 9.58%, 48.96%, 49.09%, and 55.33% on day 20) and ABTS trapping activity (from 17.38%, 38.52%, 51.19%, and 59.87% on day 1 to 12.98%, 30.30%, 35.20%, and 36.05% on day 20) for doses of 0%, 2%, 4%, and 6% of extract, respectively.

This decrease in AA is probably caused by a loss of AA of the bioactive peptides, affected by variations in amino acid content and/or changes in their sequences as a result of lower pH levels in the yogurt, which persists even during the post‐acidification period (Mashayekh et al., 2023). A further possible explanation might be the intensified interactions between milk proteins and the phenolic compounds contained in the incorporated extract (Vital et al., 2015). With this view, consumption of yogurt before the expiration of the 10 days following its production is strongly recommended in order to benefit from both the high content of live lactic acid bacteria and the high AA beneficial to the consumer's health, in particular the prevention of cardiovascular disease (Amirdivani & Baba, 2011).

On the other hand, the results obtained by the TBARS test showed a significant (*p* < .01) increase in the level of lipid peroxidation for all yogurt samples, with and without extracts, and proportionally with the time passed in the refrigerator, ranging from 0.59 to 0.74 and 1.37 mg MDA/kg as averages, respectively, obtained on the 1st, 10th, and 20th days of cold storage.

However, throughout the yogurt storage period, the highest MDA levels were obtained for plain yogurt and tended to decrease significantly (*p* < .01) with increasing PHE doses incorporated into the yogurt, ranging from 0.78 to 0.67, to 0.58, and to 0.32 mg MDA/kg on the first day, from 1.11 to 0.72, to 0.65, and to 0.48 mg MDA/kg on the 10th day, and from 1.52 to 1.42, to 1.34, and to 1.20 mg MDA/kg on the 20th for extract levels of 0%, 2%, 4%, and 6%, respectively. These results testify to the positive effect of adding PHE to attenuate this phenomenon, contributing to extended product shelf life when stored under positive refrigeration at 4°C.

Our results corroborate those advanced by several authors, reporting a significant decrease in lipid peroxidation and an improvement in stability during 20 days of cold storage by enriching yogurt with phenolic extracts derived from argel (Mohamed Ahmed et al., 2021), date palm spikelets (Almusallam et al., 2021), and seaweed (O'Sullivan et al., 2016).

### 3.4 Effect of PHE on the fatty acid composition of yogurt

Yogurt fat composition plays a significant role in determining its nutritional qualities and sensory properties (Paszczyk & Tońska, 2022). In our study, the total fat content decreased slightly (*p* > .05) during the storing period, which is likely attributable to the lipase activity released by yogurt bacteria. Lipolysis is regarded as one of the most important biochemical processes, significantly affecting the durability of many dairy foods (Alirezalu et al., 2019). It is also an important phenomenon determining characteristic flavor (Wang et al., 2023). Khalil et al. (2022) have shown a similar trend.

Furthermore, in all yogurt samples tested in this study, fluctuating levels of a number of fatty acids were observed, whether added by PHE or not, very probably resulting from the activity of yogurt‐specific bacteria that use modification of the lipid composition as an adaptive strategy against changes in environmental conditions, such as changes in acidity (Bahrami et al., 2020).

The inclusion of PHE in the yogurt formulation had no significant effect (p > 0.05) on levels of the two types of fatty acids, saturated (SFA) and unsaturated (UFA), in all yogurt samples. Nevertheless, a superiority of saturated fatty acids over unsaturated ones was detected in yogurts throughout the storage period. The unsaturation also made fatty acids more susceptible to peroxidation (Gutteridge & Halliwell, 1990), causing undesirable rangy flavors in yogurts. Similarly, Caleja et al. (2016) reported that yogurts enriched with fenugreek and chamomile extracts showed the same trend in SFA abundance relative to UFAs after 14 days of refrigeration.

In addition, incorporating PHE appears to decrease the content of short‐ and medium‐chain fatty acids (SCMFAs) in yogurt, notably butyric (C4:0), caproic (C6:0), caprylic (C8:0), capric (C10:0), and lauric (C12:0) acids. Although the SCFA reduction was statistically insignificant (*p* > .05), this is certainly beneficial both for consumer health, considering that SCMFAs are the fatty acids with the most involvement in coronary heart disease (Bianchi et al., 2017), and also for flavor, due to the decisive influence of SCMFA on the characterizing taste of the product. Caproic, caprylic, and butyric acids confer a soured taste to yogurt (Savinova et al., 2022), while the presence of capric and lauric acids may be associated with a soapy taste (Su et al., 2018).

Furthermore, fortification of yogurt with PHE appears to improve considerably (*p* < .01) the omega fat level by around 0.20% and 0.12% of total fat compared to the control on days 1 and 20 of storage, respectively, conferring beneficial effects on the health of the product given that this group of fatty acids, which the human body cannot synthesize, is known for many beneficial effects on the human heart, brain,joints, eyes, skin, and behavior (Almasi et al., 2021; Qin et al., 2017). Two hypotheses may explain this rise in omega‐3 concentrations: one relates to phenolic antioxidant properties within PHE, affording protection to this category of fatty acid from eventual hydrolysis (Bakry et al., 2019; Bouzouina et al., 2016), and the second would be the transfer into yogurt of some of the omega‐3 fats contained in the added extract, bearing in mind that peppermint leaves contain around 2.98 mg of omega‐3 fatty acids per gram of powdered plant (Savych et al., 2021). The major omega‐3 in yogurt enriched with 4% PHE is alpha‐linolenic acid (ALA, C18:3n3). Literature data reveal that an ALA‐rich diet helps to reduce the risk of developing cancer, heart disease, and acute myocardial infarction (Lin et al., 2018).

In addition, ALA is a precursor for synthesizing polyunsaturated fatty acids such as DHA (cis‐4,7,10,13,16,19‐docosahexaenoic acid, C22:6n3) and EPA (cis‐5,8,11,14,17‐eicosapentaenoic acid, C20:5n3) that perform a large array of physiological functions in the body, notably the maintenance of the shape and integrity of cell membranes, the control of numerous immune and inflammatory processes, and regulating lipid and glucose metabolism (Lutfi et al., 2022). In this study, incorporating PHE into yogurt boosted the content of ALA considerably (*p* < .01), by approximately 0.19% in comparison with the control.

Several studies suggest that high ratios of omega‐6/omega‐3 are a pathogenic factor for many diseases, including cancer, cardiovascular, inflammatory, autoimmune, and cognitive diseases, as well as obesity (Katan et al., 2021). According to the Healthy Eating Index (HEI), more healthful dietary habits are actually linked to lower omega‐6/omega‐3 ratios (Redruello‐Requejo et al., 2023).

What's more, numerous studies have shown that a high ratio of omega 6 to omega 3 is a risk factor for many diseases, including cancer, autoimmune diseases, and cardiovascular disease, namely cancer, autoimmune, cardiovascular, and inflammatory diseases, as well as obesity (Katan et al., 2021). According to the Healthy Eating Index (HEI), healthier eating habits are actually linked to lower omega‐6/omega‐3 ratios (Redruello‐Requejo et al., 2023).

In nutritional terms, the recommended omega‐6/omega‐3 ratios range from 1:1 to 5:1 (Lupette & Benning, 2020). In this study, yogurt containing 4% PHE achieved omega‐6/omega‐3 values in line with accepted standards on days 1 (4.32) and 20 (4.11), in contrast to the control, for which the ratios were 6.764 and 5.21 (Table 4).

**TABLE 4:** Fatty acid profile (fatty acid content in percent of total lipids) of yogurts formulated with peppermint hydroethanolic extract during storage.

Periods	1st day		20th day		Effect of PHE
Yogurts	Control	4% PHE	Control	4% PHE	
TFC (g/100 g)	01.84 ± 0.09	01.84 ± 00.06	01.82 ± 00.03	01.83 ± 00.05	*p* > .05
C4:0	01.49^b^ ± 00.07	01.43^b^ ± 00.04	01.64^b^ ± 00.03	01.49^b^ ± 00.04	*p* < .01
C6:0	01.29 ± 00.06	01.25 ± 00.04	01.33 ± 00.02	01.27 ± 00.03	*p* > .05
C8:0	00.98 ± 00.05	00.96 ± 00.03	01.00 ± 00.01	00.97 ± 00.03	*p* > .05
C10:0	02.56 ± 00.13	02.48 ± 00.08	02.58 ± 00.04	02.50 ± 00.07	*p* > .05
C11:0	00.05^a^ ± 00.00	00.05^a^ ± 00.00	00.04 ± 00.00	00.05^b^ ± 00.00	*p* < .01
C12:0	03.35 ± 00.17	03.26 ± 00.10	03.32 ± 00.06	03.33 ± 00.09	*p* > .05
C13:0	00.09^c^ ± 00.01	00.12^a^ ± 00.00	00.10^b^ ± 00.00	00.09^b^ ± 00.00	*p* < .05
C14:0	12.04 ± 00.6	11.77 ± 00.36	11.88 ± 00.19	11.97 ± 00.32	*p* > .05
C14:1	00.64 ± 00.03	00.62 ± 00.02	00.62 ± 00.01	00.62 ± 00.01	*p* > .05
C15:0	01.32 ± 00.07	01.30 ± 00.04	01.304 ± 00.02	01.35 ± 00.04	*p* > .05
C16:0	35.25 ± 01.75	35.21 ± 01.07	35.08 ± 00.57	35.93 ± 00.90	*p* > .05
C16:1	02.17 ± 00.11	02.25 ± 00.07	02.27 ± 00.04	02.19 ± 00.06	*p* > .05
C17:0	00.67^a^ ± 00.03	00.62^b^ ± 00.02	00.63^ab^ ± 00.01	00.50^c^ ± 00.01	*p* < .01
C18:0	08.99 ± 00.45	09.20 ± 00.28	09.14 ± 00.15	09.48 ± 00.25	*p* > .05
C18:1n9	24.17 ± 001.2	24.16 ± 00.73	24.32 ± 00.40	23.54 ± 00.62	*p* > .05
C18:2n6	02.77^a^ ± 00.14	02.56^b^ ± 00.08	02.82^a^ ± 00.05	02.67^ab^ ± 00.07	*p* < .05
C20:0	00.13^b^ ± 00.01	00.15^a^ ± 00.01	00.13^b^ ± 00.00	00.14^b^ ± 00.00	*p* < .01
C18:3n3	00.34^d^ ± 00.02	00.53^b^ ± 00.02	00.45^c^ ± 0.01	00.64^a^ ± 00.02	*p* < .01
C20:1n9	01.15^a^ ± 00.06	01.12^a^ ± 00.03	00.94^b^ ± 00.02	00.99^b^ ± 00.03	*p* < .01
C20:2	00.05^b^ ± 00.00	00.06^a^ ± 00.00	00^c^	00^c^	*p* < .01
C20:3n3	00.09^c^ ± 00.01	00.10^b^ ± 00.00	00.12^a^ ± 00.00	00.05^d^ ± 00.00	*p* < .01
C22	00.06^b^ ± 00.00	00.07^a^ ± 00.00	00.08 ± 00.00	00.08^a^ ± 00.00	*p* < .01
C20:3n6	00.15^b^ ± 00.01	00.16^ab^ ± 00.00	00.14^c^ ± 0.00	00.17^a^ ± 00.00	*p* < .01
C24:0	00.20^b^ ± 00.01	00.62^a^ ± 00.02	00.09^d^ ± 0.00	00.11^c^ ± 00.00	*p* < .01
SFA	68.46 ± 03.39	68.46 ± 02.07	68.34 ± 01.11	69.21 ± 01.82	*p* > .05
UFA	31.53 ± 01.56	31.55 ± 00.96	31.66 ± 00.51	30.86 ± 00.81	*p* > .05
SFA/UFA	02.17 ± 00.11	02.17 ± 00.07	02.16 ± 00.04	02.24 ± 00.06	*p* > .05
MUFA	28.14 ± 01.40	28.15 ± 00.85	28.15 ± 00.46	27.33 ± 00.72	*p* > .05
PUFA	03.40 ± 00.17	03.40 ± 00.10	03.52 ± 00.06	03.52 ± 00.09	*p* > .05
SMCFA	09.71 ± 00.48	09.43 ± 00.29	09.91 ± 00.16	09.55 ± 00.21	*p* > .05
LCFA	90.30 ± 04.47	95.99 ± 02.91	90.09 ± 01.46	90.49 ± 02.38	*p* > .05
ω‐3	00.43^d^ ± 00.02	00.63^b^ ± 00.02	00.57^c^ ± 00.01	00.69^a^ ± 00.02	*p* < .01
ω‐6	02.92 ± 00.14	02.71 ± 00.08	02.95 ± 00.05	02.83 ± 00.08	*p* > .05
ω‐6/ω‐3	06.76^a^ ± 00.34	04.32^c^ ± 00.13	05.21^b^ ± 00.09	04.11^c^ ± 00.11	*p* < .01

*Note*: The results are expressed as mean values and standard deviations. For each group, the number of repetitions is 03 (*n* = 03); PHE, peppermint hydroethanolic extract; *p* < .01: highly significant effect of the studied factor (mint extract content incorporated in yogurt); *p* < .05: significant effect of the studied factor; *p* > .05: non‐significant effect of the studied factor; means in the same row with different subscripts are significantly different at the 5% probability threshold.

Abbreviations: MUFA, Mono Unsaturated Fatty Acid; PUFA, Poly Unsaturated Fatty Acid; SFA, Saturated Fatty Acids; SFA/UFA, Saturated/Unsaturated Fatty Acid Ratio; SMCFA, Short and medium chain fatty acids; TFC, total fat content; UFA, Unsaturated Fatty Acids; ω‐3, Omega 3; ω‐6, Omega 6.

### 3.5 Sensory properties of PHE‐added yogurt

The development of new food formulations requires defining consumer expectations and assessing their appreciation, which involves carrying out a sensory study of the yogurt on which the commercial success of the production would be based (Benmeziane et al., 2021). In the present study, several organoleptic criteria were taken into account to assess the sensory quality of experimental yogurts, including flavor (acid taste, aftertaste, and freshness), appearance (adhesiveness, cohesiveness, and color), and odor.

In this study, yogurt with 2% extract was best accepted, even better than control yogurt on days 10 and 20 of storage, because of its slightly acidic taste derived from moderate lactic acid synthesis by *St* and *Lb*, inhibited by the antibacterial phenolic compounds present in the added extract (Wei et al., 2023).

Moreover, thanks to the presence in *Mentha piperita* leaves of volatile constituents such as menthone, menthol, limonene, and piperitone (Eftekhari et al., 2021), the PHE seems to form a perfectly harmonious combination with the aromas normally found in yogurt. (acetaldehyde, acetone, acetylone, and diacetyl), improving the product's freshness at levels of 2 and 4% PHE. However, *St* and *Lb* production of aromas and flavors seems to be reduced as a consequence of the antibacterial effect caused by high levels of the introduced phenolic extract, so yogurt with 6% PHE was placed last in terms of freshness. At this level (6% PHE), the tasting panel described the yogurt flavors as unpleasant with a very pronounced aftertaste, likely attributed to the obvious bitter flavors derived from phenolic compounds such as rosmarinic acid and flavonoids (Weimann & Heinrich, 1997).

Furthermore, yogurt rheology is mainly expressed in terms of stickiness as well as cohesiveness and is related to the amount of exopolysaccharides generated by starter cultures (Rawson & Marshall, 1997), which was shown in the present study to be less accepted as doses of incorporated extract increased. This was probably due to lower exopolysaccharide production by yogurt lactic ferments, whose growth would be slowed by the phenolic compounds of *Mentha piperita* L., which have a recognized antibacterial effect, particularly against Gram‐positive bacteria (Wei et al., 2023). However, adding 2% and 4% PHE seems to give yogurt a better smell, combining the natural aromas of yogurt with those present in mint extract, notably piperitone, limonene, menthol, and menthone (Eftekhari et al., 2021), thus forming an agreeable olfactory cocktail, while at 6%, this bacterial activity producing aromas and scents seems to be slowed down by the antibacterial effect of the added phenolic extract (Wei et al., 2023), whose olfactive power seems to have also masked that of yogurt.

**FIGURE 2:**
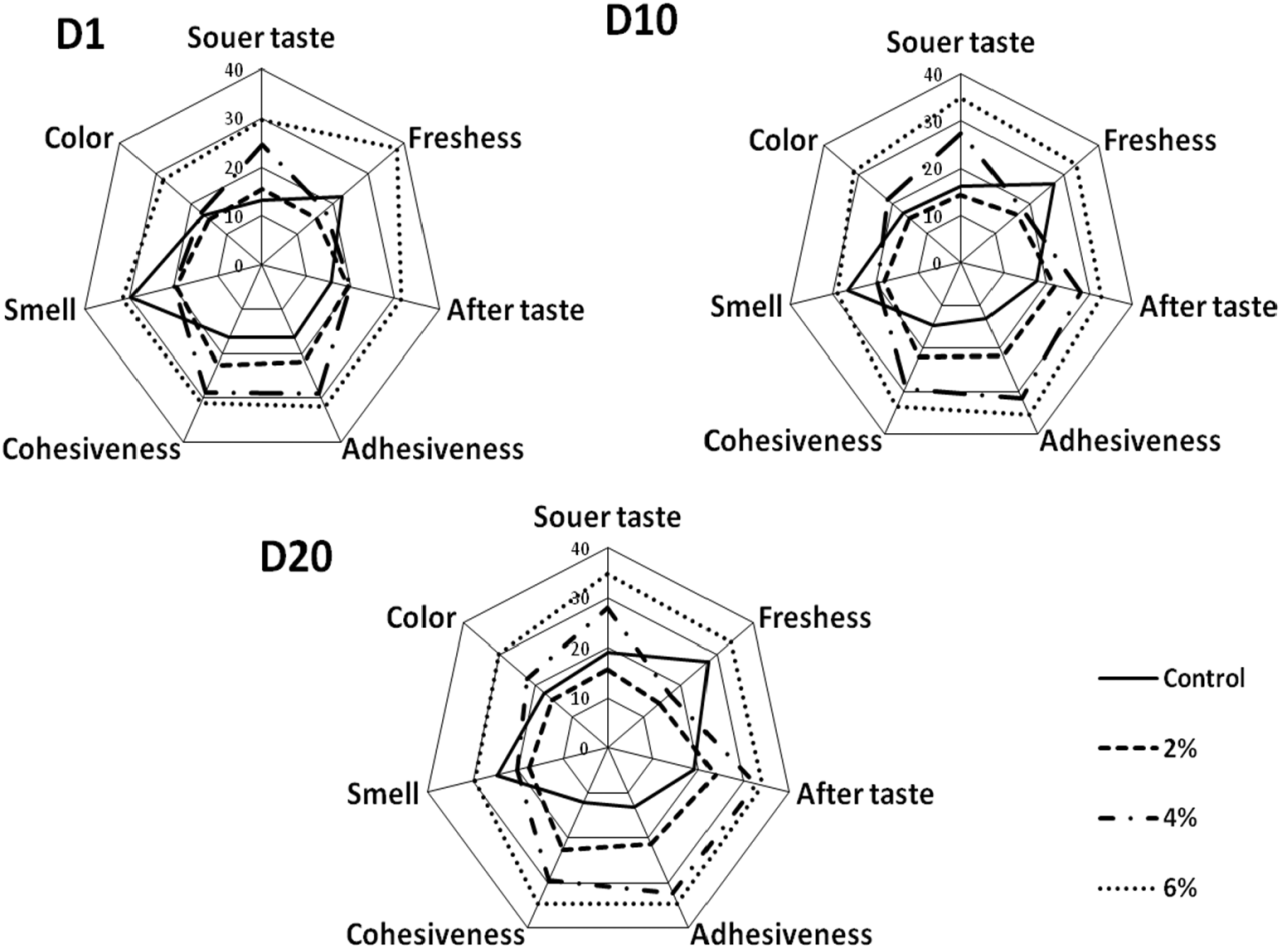
Effect of the incorporation of *Mentha piperita* hydroethanolic extract on the sensory quality of yogurt. (D1) Day 1; (D10) Day 10; (D20) Day 20.

In terms of color, yogurts supplemented with extract at 2% and 4 were well appreciated for their slightly greenish color. The color obtained at 2% was even preferred by the tasting panel to the natural whitish color of the control yogurt throughout the storage period, while yogurts with 6% extract had the lowest scores due to their intensified colors having a less pleasant appearance, certainly conferred by the chlorophyll and certain phenolic compounds contained in the extract, including anthocyanins (Farnad et al., 2014) (Figure 2).

## 4 CONCLUSION

*Mentha piperita* L. hydroethanolic extract contains a wide variety of phenolic compounds, the most abundant of which is rosmarinic acid, giving it a high antioxidant capacity. Adding PHE to yogurt, even at low doses, helps considerably to protect the lipid fraction and improve the sensory properties of the product in storage.

Compared to the control, yogurt prepared with a 4% hydroethanol extract of peppermint stands out for its high antioxidant capacity, greater resistance to lipid peroxidation, acceptable physicochemical and organoleptic quality, significantly improved α‐linolenic acid and omega‐3 values, and a ω6/ω3 ratio conforming to accepted standards. This fortified yogurt can be considered a new functional food capable of meeting the nutritional and dietary needs of consumers. This makes peppermint hydroethanolic extract an excellent choice as a natural alternative to synthetic antioxidants in the food industry.

## AUTHOR CONTRIBUTIONS

**Chafika Guemidi:** Formal analysis (equal); methodology (equal); visualization (equal); writing – original draft (equal); writing – review and editing (equal). **Djamal Ait Saada:** Conceptualization (equal); methodology (equal); project administration (equal); supervision (equal); validation (equal). **Ouiza Ait Chabane:** Formal analysis (equal); investigation (equal); methodology (equal); visualization (equal). **Mahfuz Elmastas:** Formal analysis (equal); investigation (equal); methodology (equal). **Ramazan Erenler:** Formal analysis (equal); investigation (equal); methodology (equal). **Mustafa Abdullah Yilmaz:** Formal analysis (equal); investigation (equal); methodology (equal). **Abbas Tarhan:** Formal analysis (equal); investigation (equal). **Salah Akkal:** Formal analysis (equal); investigation (equal); methodology (equal). **Haroune Khelifi:** Formal analysis (equal); investigation (equal).

## FUNDING INFORMATION

This research was not funded.

## CONFLICT OF INTEREST STATEMENT

The authors declare no conflicts of interest.

## ETHICS STATEMENT

This study does not involve any human or animal testing.

## Data Availability

Research data are not shared.
